# The Effect of Tai Chi (Bafa Wubu) Training and Artificial Intelligence-Based Movement-Precision Feedback on the Mental and Physical Outcomes of Elderly

**DOI:** 10.3390/s24196485

**Published:** 2024-10-09

**Authors:** Yuze Zhang, Haojie Li, Rui Huang

**Affiliations:** 1 School of Exercise and Health, Shanghai University of Sport, Shanghai 200438, China; zhangyuze999bsu@163.com (Y.Z.); 202121070037@mail.bnu.edu.cn (H.L.); 2 Chinese WuShu Academy, Beijing Sport University, Beijing 100084, China

**Keywords:** tai chi, movement recognition, inertial measurement units, temporal convolutional neural networks, elderly intervention

## Abstract

(1) Background: This study aims to compare the effects of AI-based exercise feedback and standard training on the physical and mental health outcomes of older adults participating in a 4-week tai chi training program. (2) Methods: Participants were divided into three groups: an AI feedback group received real-time movement accuracy feedback based on AI and inertial measurement units (IMUs), a conventional feedback group received verbal feedback from supervisors, and a control group received no feedback. All groups trained three times per week for 8 weeks. Outcome measures, including movement accuracy, balance, grip strength, quality of life, and depression, were assessed before and after the training period. (3) Results: Compared to pre-training, all three groups showed significant improvements in movement accuracy, grip strength, quality of life, and depression. Only the AI feedback group showed significant improvements in balance. In terms of movement accuracy and balance, the AI feedback group showed significantly greater improvement compared to the conventional feedback group and the control group. (4) Conclusions: Providing real-time AI-based movement feedback during tai chi training offers greater health benefits for older adults compared to standard training without feedback.

## 1. Introduction

The elderly population is rapidly increasing globally, a trend that brings about an urgent need for attention to their physical and mental health. With the increase in life expectancy and advances in medical technology, the quality of life of the elderly has become a social concern [1]. Physically, older adults often face problems such as muscle atrophy and arthritis, while psychologically, depression and anxiety often accompany them [2]. These health challenges not only affect their quality of daily life but also may lead to more serious social and economic burdens [3]. Therefore, finding effective ways to manage health is particularly important for older adults. Of particular concern is the fact that the body of older adults gradually loses vigor and elasticity as they age, leading to exercise as an important means of maintaining health [4]. However, older adults face challenges in choosing exercise programs and mastering exercise techniques and are prone to problems such as inaccurate exercise postures and inappropriate training methods, leading to poor exercise results and even injuries [5,6].

Exercise is widely recognized as an effective means of improving the health of older adults, helping to maintain muscle strength, improve cardiorespiratory fitness, and help prevent many chronic diseases [7]. However, many traditional exercises may have limitations for older adults, e.g., high-intensity exercises may be too intense and lead to injury or discomfort. Therefore, there is a need to explore low-risk, high-efficiency exercise options for older adults [8]. Tai chi, a traditional Chinese fitness exercise, is attracting more and more older adults to participate in its unique slow movements and breathing techniques. Studies have shown that tai chi practice has benefits for several aspects of health in older adults. In terms of mental health, tai chi has been shown to reduce anxiety and depressive symptoms and enhance psychological well-being [9,10]. In terms of physical function, tai chi improves balance, flexibility, and muscle strength, prevents falls, and enhances functional independence in daily living [11]. In addition, tai chi helps to improve the quality of life of older adults by promoting overall well-being through enhanced physical function and mental health status [12]. Despite the growing popularity of tai chi in the older adult population, in-depth quantitative analytical studies on its movement techniques remain relatively scarce. Previous researchers have noted that it is the lack of understanding of the accuracy and standardization of tai chi movements that may prevent older adults from fully enjoying its health benefits and even increase the risk of exercise injuries [13]. Therefore, in-depth studies on the characteristics of tai chi movements and their relationship with the health status of older adults are of great significance and can help to optimize the design of training instructions and individualized interventions. To address this challenge, inertial measurement unit (IMU) technology, introduced in recent years, provides new tools and methods for research. IMUs are able to capture real-time and precise movement data during tai chi exercise, including posture, speed, and angular changes, thus helping researchers to analyze the execution of each movement in greater detail [14]. Studies have demonstrated that human movements can be effectively identified and recorded through the use of IMUs to provide more precise and personalized training instructions for older adults, thereby optimizing their exercise performance and health outcomes [15].

Although tai chi is considered beneficial for older adults, the effectiveness of its training often relies on the accuracy and standardization of movements. In current practice, the teaching and instruction of tai chi lacks individualization and precision, resulting in the possibility that some older adults may not be able to achieve maximum health benefits. Therefore, more precise teaching methods and techniques are needed to ensure the correctness and effectiveness of older adults’ movements in tai chi training to maximize their health benefits [16].

Sensor technologies, especially inertial measurement units (IMUs), are becoming increasingly popular in sports for real-time and accurate capture of human motion data [17]. Inertial measurement units (IMUs) are favored for their small size, affordability, and portability [18,19] and are pivotal in sports applications. For example, studies have shown that activities such as running involve various factors such as body posture, speed, and angle. Traditional subjective assessment methods are prone to uncertainty. IMU technology can objectively monitor and quantitatively analyze lower limb movements during running, thus improving the accuracy and guidance of research [20]. In addition, smart bracelets equipped with IMU sensors can provide real-time feedback on exercise movements, helping users to correct their posture and improve exercise quality [21]. IMU sensors in exercise training can also monitor exercise status, detect potential health problems in a timely manner, and provide targeted interventions to improve the quality of life of older adults, thus contributing to health management and disease prevention [22]. In addition, IMU sensors help to accurately monitor and quantitatively assess tai chi movements, providing scientific insights and personalized guidance for elderly tai chi training.

Introducing modern artificial intelligence techniques into the field of tai chi training to provide precise exercise feedback and guidance for the elderly is a cutting-edge and innovative direction of current research. We use a hybrid Temporal Convolutional Network-Long Short-Term Memory (TCN-LSTM) model, which combines two mainstream techniques for temporal data processing. Temporal Convolutional Networks (TCNs) have advantages in capturing long-term dependencies and pattern recognition, while Long Short-Term Memory Networks (LSTMs) can effectively deal with long-range dependencies in temporal data [23,24]. With this hybrid model, we are able to more accurately analyze and predict the movement execution process of older adults in tai chi training. Currently, there are relatively few applications of this type of technique in the field of sports and health, especially in the accurate prediction and feedback of traditional fitness activities such as tai chi. Our study hypothesizes that AI-assisted tai chi training will result in greater improvements in physical fitness and life satisfaction among older adults compared to traditional training methods. By leveraging advanced AI technologies for real-time data collection and analysis, we aim to provide personalized and accurate fitness guidance, which not only enhances the effectiveness and safety of tai chi training but also contributes to pushing the frontiers of exercise health management. This research holds the potential to offer new possibilities and hope for improving the health and well-being of the elderly population.

## 2. Participants and Method

### 2.1. Participants

This study aimed to evaluate the impact of AI-based precise feedback tai chi on the physical and mental health of elderly individuals. Given the lack of comparable group parameters in the previous literature, G*Power (version 3.1.9.4) was used to estimate the required sample size. A medium effect size (ES = 0.30) was assumed, with a significance level of 0.05 and a desired power of 0.80 [25]. With three groups, the initial estimated sample size was 30 participants, with 10 participants per group. Considering a potential attrition rate of 30%, each group required 15 participants, totaling 45 participants. To further enhance the statistical power of the study, the sample size was increased to 20 participants per group, resulting in a total of 60 participants.

Elderly males were recruited from six communities and randomly assigned to one of three groups: the AI group (AI) receiving real-time movement accuracy feedback based on artificial intelligence and inertial measurement units (IMUs), the traditional feedback group (CF) receiving verbal feedback from supervisors, and the control group (CON) receiving no feedback.

**Figure 1:**
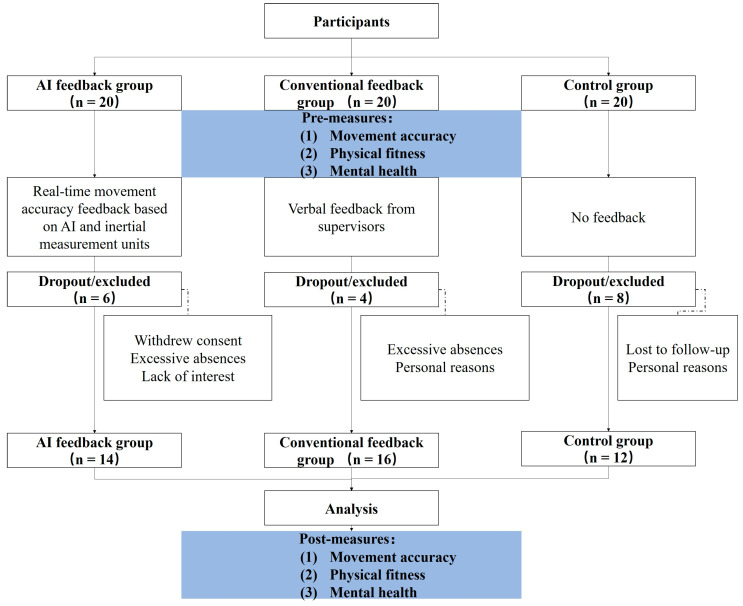
Experimental flow chart.

Inclusion criteria were as follows: (1) individuals aged 60 years and older; (2) no cognitive impairments, as determined by the Montreal Cognitive Assessment (MoCA) scale; A Montreal Cognitive Assessment (MoCA) scale score of ≥26 was classified as normal, 18–25 as mild cognitive impairment, and <18 as significant cognitive impairment [26]. Participants with scores ≥26 were selected for the study. (3) ability to follow simple instructions; (4) capability to walk without assistance; (5) normal verbal communication ability, assessed through verbal interactions during screening; (6) without tai chi training experience; (7) provision of informed consent. Exclusion criteria included acute or unstable medical conditions, severe musculoskeletal disorders, and significant difficulties in standing or walking. During the study, 18 participants withdrew for reasons detailed in Figure 1, leaving a final sample of 42 participants who completed the entire study.

All participants provided informed consent subsequent to receiving comprehensive information regarding the study’s aims, methodologies, potential risks, and benefits. The research protocol underwent a comprehensive review by the Institutional Ethics Committee at the Beijing Normal University, adhering strictly to the tenets of the Declaration of Helsinki. Following this review, the protocol was granted ethical clearance under the approval number.

### 2.2. Method

#### 2.2.1. Study Design

Participants in the AI Precision Intervention group were provided with an armband for their left upper arm to hold a smartphone equipped with an inertial measurement unit (IMU). The smartphone’s data were used by a specialized application to assess movement accuracy in real-time. Participants received supplementary, customized guidance or encouragement based on real-time feedback.

The Conventional Feedback group received real-time feedback from a supervisor during each training session. Supervisors were instructed to closely observe the participants’ movements and provide corrections, guidance, or encouragement for any incorrect movements observed.

The control group engaged in regular training without receiving any form of feedback.

All three groups participated in an 8-week tai chi intervention program, with sessions conducted three times per week. Each session consisted of a structured routine beginning with a 5 min warm-up, followed by 30 min of continuous tai chi practice, and concluding with a 5 min cool-down period. The accuracy of each movement, along with balance, grip strength, quality of life, and depression indicators, were assessed for all three groups before and after the intervention period.

#### 2.2.2. Tai Chi (Bafa Wubu) Exercise

**Figure 2:**
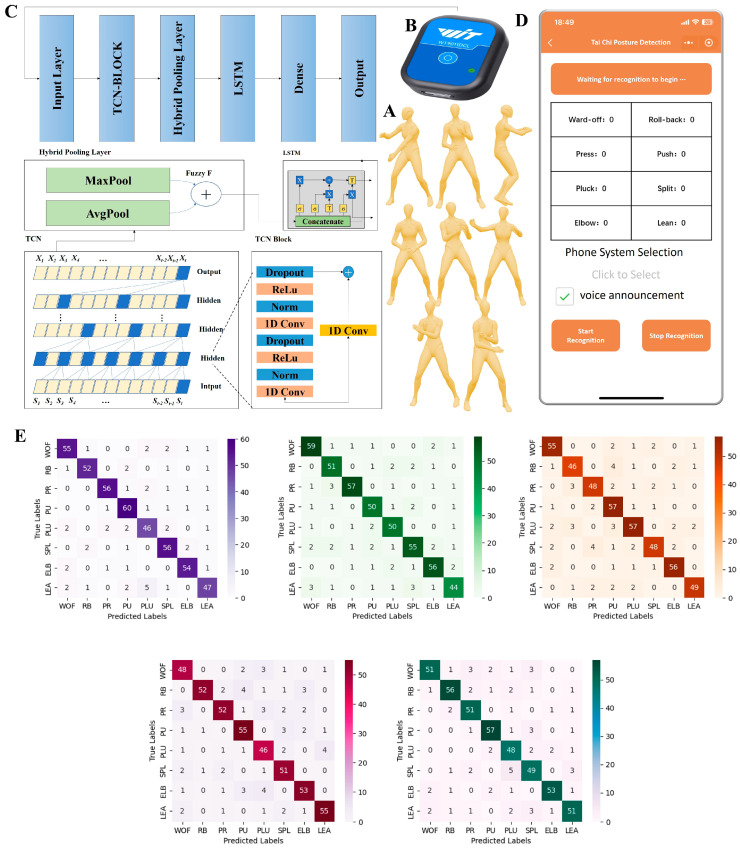
Technological process for implementing AI feedback-based tai chi interventions; (**A**) the “Eight Techniques and Five Steps” (Taiji Bāfǎ Wǔbù); (**B**) IMU sensors for collecting motion data; (**C**) architecture of the TCN-LSTM neural network; (**D**) deployment of motion recognition algorithms on WeChat applets; (**E**) confusion matrix for five-fold cross-validation of models. Abbreviations: TCN: temporal convolutional neural network; LSTM: long- and short-term memory networks; 1D Conv: one-dimensional causal convolutional layer; WOF: ward-off; RB: roll-back; PR: press; PU: push; PLU: pluck; SPL: split; ELB: elbow; LEA: lean.

The “Eight Techniques and Five Steps” (TaiChi Bāfǎ Wǔbù) form of tai chi was developed to better promote, disseminate, and popularize tai chi. It is systematically derived from the most essential techniques across existing tai chi schools. This form focuses on eight fundamental hand techniques—ward-off (Péng), roll-back (Lǚ), press (Jǐ), push (Àn), pluck (Cǎi), split (Liè), elbow (Zhǒu), and lean (Kào)—and five essential steps—advance (Jìn), retreat (Tuì), look left (Gù), look right (Pàn), and central equilibrium (Dìng) (see Figure 2A). These elements have been meticulously refined and organized to create a form that is structurally simple, reasonably comprehensive, rich in content, and easy to learn and practice [16].

Given that steps are generally performed in coordination with hand techniques and that the hand techniques themselves are more complex and central to the form, the primary focus of this study is on assessing the accuracy of the eight hand techniques. By controlling and ensuring the accuracy of these techniques, we aim to achieve overall quality control in tai chi practice. The simplified structure and intrinsic richness of the “Eight Techniques and Five Steps” make it an ideal form for both beginners and advanced practitioners, facilitating widespread adoption and effective practice.

#### 2.2.3. AI Feedback Implementation Process

A hybrid Temporal Convolutional Network-Long Short-Term Memory (TCN-LSTM) neural network model was developed to analyze the movement data (see Figure 2C). This model, trained on a large dataset, achieved high accuracy rates ranging from 81% to 92% in motion prediction (see Figure 2E and Table S1).

The trained model was deployed on a WeChat applet to provide real-time feedback. The applet facilitated the motion detection process by transmitting data to a server hosted on Tencent Cloud (see Figure 2D). On the server, the TCN model classified the movements and provided accuracy metrics. This setup enabled participants to receive immediate feedback during their tai chi practice. All data were securely stored on the cloud server for further analysis, ensuring both the integrity and confidentiality of the information collected.

See the Supplementary Materials for the training dataset, model construction, and model performance evaluation.

#### 2.2.4. Outcome Measures

This research used a broad array of health outcome measures to assess various health aspects in elderly participants, including accuracy of movements, balance ability, grip strength, quality of life, and levels of depression.

To evaluate movement accuracy, the process was divided into a pre-test and a post-test. In both stages, participants first watched a full demonstration of a fixed sequence of eight tai chi techniques (Bāfǎ) performed by a professional instructor. The sequence and order of movements were consistent across both tests to ensure standardized evaluation. After the demonstration, participants were asked to replicate the movements as closely as possible based on what they had observed. At the start of the replication, a specialized application was activated to automatically analyze the participants’ movements in real-time. The application classified each movement based on predefined categories and continuously monitored the execution. The accuracy rate for each movement was determined by comparing the expected number of correct movements with the actual movements performed by the participants. This allowed for a precise evaluation of how closely their performance matched the ideal standard. All detailed procedures and analysis methods are provided in the Supplementary Materials.

Balance ability was assessed through the one-leg stand test with eyes closed, which is a recognized method for evaluating postural stability. Each participant used a balance meter (Model HK6000, Hengkang Jiaye, Guangzhou, China) to obtain accurate and standardized measurements. Timing started automatically when the participant lifted one foot and stood on the balance meter with the other foot. It stopped when the participant placed both feet back on the plate. The duration (in seconds) of each stance was recorded, and the average result from three attempts was used for analysis.

For grip strength, an important indicator of upper limb strength and overall muscular health, a hand dynamometer (Model HK6800, Hengkang Jiaye, Guangzhou, China) was employed. Participants stood with their arms naturally down by their sides, palms facing inward, and performed a single-grip test with their dominant hand. The mean value of three successive trials was recorded to ensure the reliability of the test results.

Quality of life was measured using the 12-Item Short Form Survey (SF-12). This widely adopted instrument, which succinctly captures crucial physical and mental health dimensions, provides a comprehensive view of an individual’s health-related quality of life. Previous studies have thoroughly validated the psychometric properties of the Chinese version of the SF-12, ensuring its scientific rigor and accuracy in the assessment process. In our study, the SF-12 showed an internal consistency reliability with a Cronbach’s alpha of 0.855.

The Beck Depression Inventory (BDI) was used to screen for Psychological Depression, measuring the presence and severity of depressive symptoms. The Chinese version of the BDI has also demonstrated favorable psychometric validation in prior research, rendering it a reliable instrument for assessing depressive symptoms in the elderly population. The BDI in this study demonstrated high internal consistency reliability, with a Cronbach’s alpha of 0.873.

### 2.3. Statistical Analysis

Descriptive statistics were conducted to summarize the data, and all data passed the normality tests. A mixed-effects analysis of variance (ANOVA) was employed to examine the effects of between-subject factors (group assignments: AI, CF, CON) and within-subject factors (time: pre-intervention and post-intervention) on the outcome variables. Statistical analyses were performed using IBM SPSS 26.0 Statistics software. A *p*-value of less than 0.05 was considered to indicate statistical significance.

## 3. Results

The results showed no significant differences between the AI group, CF group, and CON group across all metrics before the intervention.

Ward-off Accuracy: There was an interaction effect between group and time (*p* = 0.003, η^2^_p_ = 0.263). Simple effect analysis for time revealed that the AI group, CF group, and CON group all showed significant improvement compared to pre-intervention. Simple effect analysis for the group indicated that post-intervention, the AI group was significantly higher than the CF group and CON group (*p* = 0.013; *p* = 0.002) (see Table 1).

Accuracy of Other Movements: There was no interaction effect between group and time for other movements. Simple effect analysis for time showed that the AI group, CF group, and CON group all significantly improved compared to pre-intervention. Simple effect analysis for the group indicated that post-intervention, the AI group’s accuracy in roll-back was significantly higher than that of the CF group and CON group (*p* = 0.007; *p* = 0.006); the AI group’s accuracy in pluck was significantly higher than that of the CF group and CON group (*p* = 0.039; *p* = 0.001) (see Table 1).

**Figure 3:**
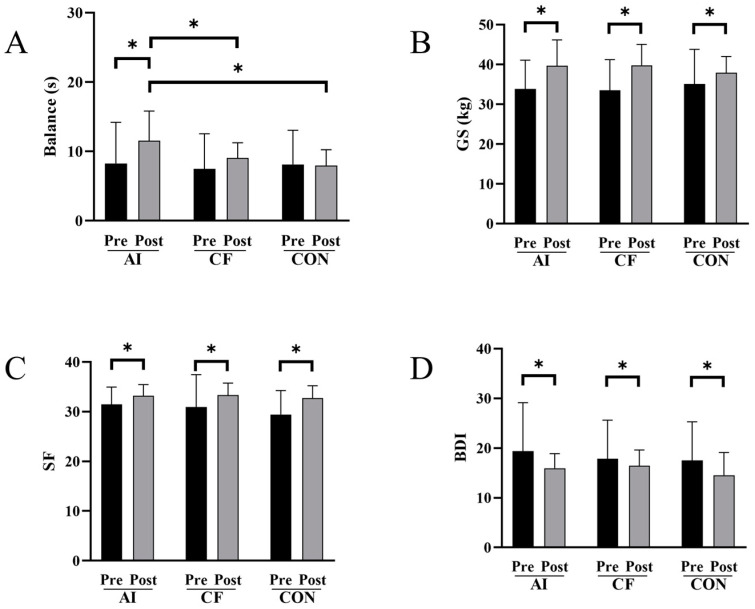
Fitness and mental health indicators before and after the intervention. (**A**) Balance; (**B**) grip strength; (**C**) SF-12; (**D**) BDI; Abbreviations: GS: grip strength; SF-12: 12-Item Short Form Survey; BDI: Beck Depression Inventory; AI: AI feedback group; CF: conventional feedback group; CON: control group. * indicates significant differences.

Balance: There was no interaction effect between group and time. Simple effect analysis for time revealed that only the AI group showed significant improvement compared to pre-intervention. Simple effect analysis for the group indicated that post-intervention, the AI group was significantly higher than the CF group and CON group (*p* = 0.037; *p* = 0.015) (see Table 1 and Figure 3A).

Other Fitness and Mental Health Indicators: There was no interaction effect between group and time. Simple effect analysis for time revealed that the AI group, CF group, and CON group all showed significant improvement compared to pre-intervention (see Table 1 and Figure 3A–D). Post-intervention simple effect analysis for the group showed no significant differences.

## 4. Discussion

Our study found that the AI group, CF group, and control group had significantly higher precision in all movements after the intervention. The simple effect analysis by group showed that the AI group had significantly higher precision in the ward-off maneuver than the CF and control groups after the intervention. The AI, CF, and control groups had significantly higher precision in all other maneuvers. Specifically, in roll-back and pluck maneuvers, the precision of the AI group was significantly higher than that of the CF and control groups. This suggests that the AI technique was not only effective in the ward-off maneuver but also showed significant advantages in other tai chi maneuvers. These findings suggest that the precise feedback of AI technology can effectively enhance the execution accuracy of tai chi movements in older adults, thereby improving their motor skills and body control. Studies have shown that tai chi not only improves flexibility and muscle strength but also helps to improve balance and mental health status, such as reducing anxiety and depressive symptoms [27]. However, motor skills and precision of movement execution in older adults tend to decline due to age-related physiological changes, which limits their effectiveness and safety in fitness activities. The effectiveness of tai chi in older adults’ fitness is further strengthened by the results found in this study, namely that AI technology can enhance the precision of tai chi movements. Specifically, the real-time feedback provided by AI can help older adults more accurately understand and execute complex tai chi movements such as ward-off, roll-back, and pluck. This not only helps them to better master the movement skills in the program, but also improves their movement coordination and body control [28].

Additionally, our study found no significant interaction effect between group and time in terms of balance ability, but a simple effects analysis of time showed that only the AI group showed a significant improvement in balance ability after the intervention. The simple effects analysis for groups showed that the AI group showed significantly higher balance ability than the CF and control groups after the intervention. This suggests that the motor precision feedback of AI technology not only improves the accuracy of tai chi movements but also helps to improve the balance control ability of older adults, which is important for the prevention of accidents such as falls [29]. As a very suitable form of exercise for older adults, tai chi focuses on body posture, balance, and fluid movements. However, due to the diversity of body states and age-related physiological changes in older adults, they are often challenged with inaccurate movements and decreased balance control when practicing tai chi. These problems not only affect the effectiveness of the exercise but also increase the risk of falls and other accidents [30]. One of the key findings of this study was that the AI technology’s movement precision feedback significantly improved older adults’ movement accuracy in tai chi. Specifically, the AI group’s post-intervention ward-off movement performance was significantly better than that of the CF group and the control group, reflecting the ability of AI technology to help older adults more finely master and execute complex movement requirements. For example, through real-time feedback and adjustment, AI technology can prompt older adults to adjust their posture, strengthen core stability, and accurately control the center of gravity of the body, thereby improving the fluidity and execution of movements [31]. In addition, it was found that the AI group also showed significant improvement in balance ability, although there was no significant interaction effect between group and time. The intervention of AI technology significantly improved the balance control ability of older adults, which is important for the prevention of accidents such as falls. The balance ability of older adults is not only related to the safety of daily life but also directly affects their quality of life and autonomy [32,33]. Through tai chi training combined with AI technology, older adults are able to enhance core stability and improve postural control, thus moving more freely and safely in their daily activities. Further research could explore the long-term effects of different AI feedback systems on the motor performance and quality of life of older adults. In addition, the specific effects of different types and frequencies of AI feedback on tai chi learning outcomes could be compared to optimize the design of future health promotion strategies and fitness programs for older adults.

On the other hand, there was no significant interaction effect between group and time for other health and mental health indicators, suggesting that while AI technology had a significant effect on movement accuracy and balance, improvements in mental health indicators may be influenced by a combination of factors. This study found no significant effects of AI technology on other health and mental health indicators. Although all groups showed improvements after the intervention, these improvements did not reach significant differences between groups. This may indicate that the health benefits of tai chi, as a comprehensive activity, are influenced by a variety of factors, including the overall health of the participants, the comprehensiveness of the program design, and the individual’s adaptability to exercise [34]. Future research could further explore how to optimize the application of AI techniques to more comprehensively promote health and quality of life in older adults. For example, additional physiological monitoring techniques, such as heart rate variability, sleep quality, and other metrics, could be incorporated to assess the potential of AI technology in comprehensive health promotion. In addition, long-term follow-up and intervention studies can be conducted to gain insight into the long-term impact of AI technologies on the health of older adults in order to develop more effective health management strategies and intervention programs.

Limitations of the study. The group receiving the feedback may have been more motivated by their participation in advanced technology and important medical care. This may have led to their greater involvement, which in turn presented a more significant improvement in the measurements [35]. Therefore, the possibility that feedback, regardless of its form, may bring good results in itself because of its reinforcing effect and authority cannot be completely ruled out. Although the present study demonstrated the potential of AI technology to improve movement accuracy and balance, this result could also be influenced by the participants’ own motivations and expectations. Future studies could consider introducing more control groups to further validate the specific role of AI technology in health promotion for older adults. Additionally, while pre- and post-training evaluations provide valuable insights, the current study did not capture the rate of improvement differences between groups over time. Future studies could include repeated measurements to explore whether there are rapid improvements associated with specific feedback methods, such as AI-assisted training, and to better understand the feedback’s immediate and long-term effects.

## 5. Conclusions

It can be concluded from the results of this study that AI technology demonstrates significant advantages in tai chi practice for older adults, especially in improving movement accuracy and balance. This study showed that the AI group outperformed the control group and the traditional feedback group in the execution accuracy of several tai chi movements, especially in the ward-off, roll-back, and pluck movements. This suggests that real-time accurate feedback from AI technology not only helps older adults master complex movement skills but also significantly improves their balance control, thus providing important support for preventing accidents such as falls. Although the effects on other health and mental health indicators are not yet evident, these results still provide useful insights for optimizing the application of AI technology and enhancing the overall health of older adults in the future. Therefore, further research and long-term follow-up are necessary to fully understand and maximize the potential of AI technology in health promotion for older adults.

## Figures and Tables

**Table 1:** Indicators of health outcomes before and after the intervention.

	AI Feedback Group (*n* = 14)			Conventional Feedback Group (*n* = 16)			Control Group (*n* = 12)			Interaction	
	Pre	Post	*p*	Pre	Post	*p*	Pre	Post	*p*	*p*	η^2^_p_
Ward-off (%)	9.885 ± 3.708	82.221 ± 6.231	0.000	10.844 ± 4.014	77.111 ± 4.167 ^a^	0.000	11.599 ± 2.660	75.649 ± 2.404 ^ab^	0.000	0.003	0.263
Roll-back (%)	13.606 ± 6.448	82.197 ± 7.812	0.000	14.249 ± 6.968	75.875 ± 3.696 ^a^	0.000	13.217 ± 6.766	75.309 ± 2.476 ^ab^	0.000	0.205	0.078
Press (%)	24.850 ± 6.148	79.445 ± 3.619	0.000	24.272 ± 7.142	78.841 ± 3.569	0.000	21.736 ± 6.421	79.355 ± 2.445	0.000	0.441	0.041
Push (%)	19.737 ± 9.074	79.805 ± 6.051	0.000	17.888 ± 9.097	78.046 ± 8.021	0.000	15.213 ± 7.367	76.970 ± 9.103	0.000	0.899	0.005
Pluck (%)	14.090 ± 7.006	87.526 ± 5.692	0.000	12.343 ± 7.999	80.033 ± 5.50 ^a^	0.000	11.695 ± 8.145	75.032 ± 11.770 ^ab^	0.000	0.275	0.064
Split (%)	18.209 ± 5.252	77.239 ± 8.304	0.000	20.802 ± 4.708	77.297 ± 10.313	0.000	16.610 ± 6.450	74.174 ± 5.405	0.000	0.812	0.011
Elbow (%)	18.209 ± 5.252	81.392 ± 11.228	0.000	20.802 ± 4.708	77.779 ± 10.336	0.000	16.610 ± 6.450	75.487 ± 5.230	0.000	0.251	0.068
Lean (%)	18.209 ± 5.252	88.409 ± 7.391	0.000	20.802 ± 4.708	82.694 ± 7.546	0.000	16.610 ± 6.450	83.907 ± 10.624	0.000	0.121	0.103
Balance(s)	8.270 ± 5.930	11.545 ± 4.260	0.047	7.470 ± 5.075	9.071 ± 2.158 ^a^	0.330	8.120 ± 4.924	7.966 ± 4.269 ^ab^	0.935	0.414	0.044
Grip strength (kg)	33.875 ± 7.231	39.706 ± 6.480	0.028	33.539 ± 7.676	39.816 ± 5.240	0.012	35.110 ± 8.711	37.928 ± 4.091	0.046	0.607	0.025
SF-12	31.493 ± 3.472	33.220 ± 2.224	0.049	30.966 ± 6.480	33.361 ± 2.383	0.000	29.415 ± 4.830	32.752 ± 2.453	0.035	0.742	0.015
BDI	19.390 ± 9.765	15.935 ± 2.962	0.005	17.848 ± 7.754	16.451 ± 3.155	0.045	17.523 ± 7.773	14.565 ± 4.562	0.010	0.785	0.012

Note: Abbreviations: SF-12, 12-Item Short Form Survey; BDI: Beck Depression Inventory; ES: Effect size. ^a^ indicates a significant difference when compared with the AI feedback group, while the symbol. ^b^ indicates a significant difference when compared with the conventional feedback group.

## Data Availability

The data that support the findings of this study are available on request from the corresponding author.

## Supplementary Materials

The following supporting information can be downloaded at: https://www.mdpi.com/article/10.3390/s24196485/s1, Table S1: Five-fold cross-validation of models.
