# Supplementary material for: The Effect of Tai Chi (Bafa Wubu) Training and Artificial Intelligence-Based Movement-Precision Feedback on the Mental and Physical Outcomes of Elderly
Source: Sensors (Basel). 2024 Oct 9;24(19):6485. doi: 10.3390/s24196485 (PMC11479303; doi:10.3390/s24196485)
Supplement: Supplementary file 1 [file sensors-24-06485-s001.zip › sensors-3164509-supplementary.pdf]

Supplementary material

Training dataset

The dataset utilized in this study was derived from a cohort of 20 proficient Tai Chi practitioners, all aged 50 years, to ensure a rich and varied source of motion data. The participants were selected based on their extensive experience and mastery of Tai Chi movements, which guarantees the dataset reflects a high level of proficiency. Data

Each participant performed a standardized set of Tai Chi movements under controlled conditions, ensuring consistency and accuracy in data capture. The data were collected using advanced motion-capture technology, which allowed for the precise recording of each movement's trajectory and execution. Dataset Composition:

The dataset comprises a total of 2,400 individual movement instances, meticulously categorized into 8 distinct classes of Tai Chi movements. Each class represents a different movement within the Tai Chi repertoire, with an equal distribution of 300 instances per class, ensuring a balanced dataset for training purposes.

IMU Data Acquisition and Processing

The WitMotion Bluetooth 2.0 Six-Axis Accelerometer-Gyroscope-Pitch Roll Yaw Angle Sensor was utilized in this study, affixed to the participants' left upper arms to capture detailed motion data during the Bafa Wubu routine. This raw data from the Inertial Measurement Unit (IMU) was rigorously recorded to provide a comprehensive overview of motion dynamics.

To eliminate high-frequency noise, the raw IMU signals were processed with a low-pass filter set at a cutoff frequency of 5 Hz. This filtering preserved relevant movement information while discarding unwanted noise. The filtered IMU data were then down-sampled to 50 Hz, aligning with the sampling rates of many smartphones, thereby optimizing the model for mobile platform deployment and enhancing the technology’s accessibility and applications.

For further analysis, the filtered data were segmented into 2-second windows, with a 50% overlap to ensure adequate context for movement recognition and account for transitions. This segmentation facilitated the extraction of meaningful and temporally coherent features from the continuous data stream, which were then used for movement classification by the neural network. Correct classifications indicated proper execution of movements, while misclassifications highlighted errors in execution.

Neural Network Construction

An enhanced TCN-LSTM neural network model was constructed for this study. The model architecture is detailed as follows:

- **Input Layer**: Incorporates raw IMU data.
- **Temporal Convolutional Network (TCN)**: Consists of four temporal blocks, each containing two dilated convolution layers, batch normalization layers, non-linear activation layers, and normalization layers. Dilated convolutions extend the receptive field exponentially while maintaining the same input-output size, enabling the model to make predictions based on longer sequences. Residual connections help in cross-layer transmission, integrating high-level features with lower-level features, mitigating vanishing or exploding gradient issues, and reducing model complexity.
- **Long Short-Term Memory (LSTM) Network**: Includes a memory block with cell state, input gate, output gate, and forget gate. These mechanisms enable LSTM to capture long-term dependencies in time-series data by regulating the amount of information stored, forgotten, or outputted at each step.
- **Hybrid Pooling Layer**: Implements max pooling and average pooling to reduce model complexity, accelerate training, and enhance performance. Feature fusion operations effectively integrate features from these pooling operations.
- **Output Layer**: A fully connected layer that synthesizes the learned features to classify Tai Chi movements, providing accuracy metrics for each movement.

Deployment on Mobile Devices

WeChat Applet Deployment: Tencent Holdings Ltd. developed the WeChat applet, which features a user interface allowing users to select their operating system (iOS or Android). Upon initiating the motion detection process, the applet sends real-time motion detection data to the server at one-second intervals.

Server-Side Deployment: The server, hosted on Tencent Cloud by Tencent Holdings Ltd., employs a Python-based Flask framework for data reception and transmission. This framework also facilitates the deployment of the TCN-LSTM model for motion detection and real-time computation. An incrementing function counts the detected movements once identified by the model.

Participants start the mobile application by inputting the required information, enabling the WeChat applet to recognize and count Tai Chi movements in real-time.

In the present study, to ensure the robustness and generalizability of the model, we employed a 5-fold cross-validation methodology to assess the performance of the TCN-LSTM (Temporal Convolutional Neural Network with Long Short-Term Memory) model. Below is an elucidation of the 5-fold cross-validation process and the evaluation metrics utilized:

5-Fold Cross-Validation

5-Fold cross-validation is a statistical technique used to evaluate the performance of a model, particularly in scenarios where the dataset is limited. The dataset is evenly partitioned into five segments, or folds. In each iteration, one fold is retained as the validation set (for testing the model), while the remaining four folds (80% of the data) serve as the training set (for training the model). This process is reiterated five times, with a different fold designated as the validation set in each iteration, thereby ensuring that every data point is utilized for both training and validation. Ultimately, the evaluation outcomes from the five iterations are averaged to obtain an estimate of the model's performance.

Evaluation Metrics

1. Accuracy: This metric measures the proportion of correct predictions made by the model, encompassing both true positives and true negatives relative to the total number of predictions. It provides a rapid overview of the model's overall performance.

$$\text{Accuracy} = \frac{\text{TP} + \text{TN}}{\text{Total examples}}$$

2. Precision: This metric evaluates the proportion of instances predicted as positive that are actually positive, i.e., the ratio of true positives to the sum of true positives and false positives. It focuses on the model's ability to minimize false positives.

$$\text{Precision} = \frac{\text{TP}}{\text{TP} + \text{FP}}$$

3. Recall (Sensitivity): Also known as the true positive rate, this metric measures the proportion of actual positives that are correctly identified by the model, i.e., the ratio of true positives to the sum of true positives and false negatives. It emphasizes the model's capability to capture all positive instances.

$$\text{Recall} = \frac{\text{TP}}{\text{TP} + \text{FN}}$$

4. F1 Score: This score is the harmonic mean of precision and recall, balancing the two in a single metric, which is particularly useful in cases of class imbalance. The F1 Score is a comprehensive indicator of the model's precision and recall.

$$F1 = 2 \times \frac{\text{Precision} \times \text{Recall}}{\text{Precision} + \text{Recall}}$$

Where:
- TP (True Positives) is the count of instances correctly predicted as positive.
- TN (True Negatives) is the count of instances correctly predicted as negative.
- FP (False Positives) is the count of instances incorrectly predicted as positive.
- FN (False Negatives) is the count of instances incorrectly predicted as negative.

These evaluation metrics provide a comprehensive understanding of the TCN-LSTM model's performance across different dimensions, including its ability to make correct predictions, avoid false positives, and capture all positive categories. The 5-fold cross-validation ensures the reliability of these metrics and the model's generalizability.

Table S1 Five folds cross-validation of models

**Class**	**Metric**	**Fold 1**	**Fold 2**	**Fold 3**	**Fold 4**	**Fold 5**	**Average**
**WOF**	Accuracy	0.89	0.91	0.9	0.87	0.84	0.88
	Precision	0.89	0.88	0.87	0.83	0.91	0.87
	Recall	0.89	0.91	0.9	0.87	0.84	0.88
	F1 Score	0.89	0.89	0.89	0.85	0.87	0.87
**RB**	Accuracy	0.91	0.89	0.84	0.83	0.88	0.85
	Precision	0.93	0.86	0.87	0.96	0.9	0.9
	Recall	0.91	0.89	0.84	0.83	0.88	0.85
	F1 Score	0.92	0.88	0.85	0.89	0.89	0.88
**PR**	Accuracy	0.9	0.89	0.84	0.83	0.91	0.85
	Precision	0.93	0.93	0.86	0.88	0.85	0.89
	Recall	0.9	0.89	0.84	0.83	0.91	0.85
	F1 Score	0.92	0.91	0.85	0.85	0.88	0.89
**PU**	Accuracy	0.92	0.89	0.92	0.87	0.9	0.9
	Precision	0.87	0.86	0.79	0.83	0.89	0.83
	Recall	0.92	0.89	0.92	0.87	0.9	0.9
	F1 Score	0.9	0.88	0.85	0.85	0.9	0.87
**PLU**	Accuracy	0.84	0.91	0.83	0.84	0.87	0.84
	Precision	0.82	0.86	0.86	0.78	0.79	0.82
	Recall	0.84	0.91	0.83	0.84	0.87	0.84
	F1 Score	0.83	0.88	0.84	0.81	0.83	0.82
**SPL**	Accuracy	0.9	0.83	0.81	0.89	0.82	0.85
	Precision	0.88	0.87	0.89	0.82	0.8	0.85
	Recall	0.9	0.83	0.81	0.89	0.82	0.85
	F1 Score	0.89	0.85	0.85	0.86	0.81	0.83
**ELB**	Accuracy	0.92	0.89	0.92	0.85	0.88	0.89
	Precision	0.92	0.89	0.89	0.87	0.93	0.9
	Recall	0.92	0.89	0.92	0.85	0.88	0.89
	F1 Score	0.92	0.89	0.9	0.86	0.91	0.89

**LEA**	Accuracy	0.81	0.81	0.88	0.89	0.84	0.85
	Precision	0.87	0.86	0.92	0.9	0.86	0.87
	Recall	0.81	0.81	0.88	0.89	0.84	0.85
	F1 Score	0.84	0.84	0.9	0.89	0.85	0.86

Note: Abbreviations: WOF: ward-off; RB: roll-back; PR: press; PU: push; PLU: pluck; SPL: split; ELB: elbow; LEA: lean.
